# Motorized 2–3 wheelers death rates over a decade: a global study

**DOI:** 10.1186/s13017-022-00412-4

**Published:** 2022-01-26

**Authors:** Yasin J. Yasin, Michal Grivna, Fikri M. Abu-Zidan

**Affiliations:** 1 Institute of Public Health, College of Medicine and Health Sciences, UAE University, Al-Ain, United Arab Emirates; 2 Department of Environmental Health and Behavioral Sciences, School of Public Health, College of Health Sciences, Mekelle University, Mekelle, Ethiopia; 3 Department of Public Health and Preventive Medicine, Second Faculty of Medicine, Charles University, Prague, Czech Republic; 4 Department of Surgery, College of Medicine and Health Sciences, UAE University, Al-Ain, United Arab Emirates

**Keywords:** Global, 2–3 Wheelers, Motorcycle, Death, Road traffic collision, Road safety

## Abstract

**Background:**

Motorized 2–3-wheelers-related death is high due to the exposed body of the driver/passenger and the high speed. The United Nation (UN) Decade of Action for road safety aimed to reduce road traffic deaths by 50% by the year 2020. We aimed to study the factors affecting the death rates of motorized 2–3 wheelers injured victims and whether the reduction in the death rates has met the UN target.

**Methods:**

Data were retrieved from the WHO Global Status Reports on Road Safety published over 2009 to 2018 which covered the years of 2007 to 2016. Studied variables included motorized 2–3 wheelers death rates, percentage of helmet-wearing rate, helmet law enforcement, speed law enforcement, gross national income per capita, vehicles/person ratio, and motorized 2–3 wheelers/person ratio. A mixed linear model was used to define factors affecting the change of motorized 2–3 wheelers death rates over time.

**Results:**

The global mean motorized 2–3 wheelers death rates increased from 2.37/100,000 population to 3.23/100,000 population over the studied decade (a relative ratio of 1.36) which was not statistically significant. Factors that affected mortality included GNI (*p* = 0.025), motorized 2–3 wheelers per person ratio (*p* < 0.0001), percentage of helmet wearing rate (*p* = 0.046), and the interaction between vehicle/person ratio and motorized 2–3 wheelers/person ratio (*p* = 0.016). There was a significant increase in the death rates over time in the low-income countries (a relative ratio of 2.52, *p* = 0.019, Friedman test), and middle-income countries (a relative ratio of 1.46, *p* < 0.0001, Friedman test), compared with a significant decrease in the high-income countries (a relative ratio of 0.72, *p* < 0.0001, Friedman test).

**Conclusions:**

Global mortality of motorized 2–3 wheelers has increased by a relative ratio of 1.36 over a recent decade. The UN target of reducing death was not met. The increase was related to the increase in motorized 2–3 wheelers per person ratio and economic inequity which has to be addressed globally. The economic global gap significantly impacts the mortality rates of motorized 2–3 wheelers.

## Introduction

Over the last decade, more than 13 million people died from road traffic collisions (RTCs) [1]. Around 2 billion vehicles are used globally of which 30% are motorized 2–3 wheelers [1–3]. Low- and middle-income countries (LMICs) use 88% of these wheelers of which 75% are in Southeast Asia [3]. There is a rapid increase in the use of motorized 2–3 wheelers worldwide because of their availability, flexibility, and affordability with the highest growth rate is in Southeast Asia (39%) [3–6]. Riding a motorized 2–3 wheeler is very risky because of the high speed and the exposed bodies of the driver and passenger. The risk of death from a crash is 28 to 34 times higher than for a car occupant [7, 8]. A quarter of road traffic deaths involve motorized 2–3 wheelers, with approximately 3.2 million deaths over the past decade [1, 3, 9, 10].

In response to this growing burden, the United Nations (UN) approved a Global Plan for the Decade of Action for Road Safety 2011–2020 aiming to reduce road deaths by half in 2020 [11]. Continuous evaluation of the global changes is essential to find whether the action plan is properly progressing [12]. The World Health Organization (WHO) Global Status Reports on Road Safety plays an important role in this evaluation [1, 9, 10, 13]. Although previous studies have examined the factors affecting the global motorized 2–3-wheeler-related deaths, they neither used repeated time global data nor examined the factors affecting the change over time [14]. Thus, we aimed to study the factors affecting the death rates of motorized 2–3 wheeler injured victims over time and whether the reduction in death rates has met the UN target.

## Methods

### Ethical consideration

Data used in this study are publicly available data from the WHO Global Status Report on Road Safety and do not need approval from a Human Research Ethics Committee.

### Definition of 2–3 wheelers

Motorized 2–3 wheelers refers to powered two- and three wheelers [3]. According to the WHO definition, “motorized 2–3 wheelers or powered two- and three wheelers (PTWs) are motor-operated two- and three-wheeled vehicles, powered by either a combustion engine or rechargeable batteries.” These included motorcycles, scooters, e-bikes, tricycles motor-rickshaws, or e-rickshaws [3].

### Data collection

Data were retrieved from the WHO Global Status Reports on Road Safety for years 2007, 2010, 2013, and 2016, which were published in 2009, 2013, 2015, and 2018, respectively [1, 9, 10, 13]. These reports had data on 178, 182, 180, and 175 countries, respectively, with complete data on motorized 2–3 wheelers death on 115 (64.6%), 123 (67.6%), 117 (65.0%), and 122 (69.7%) countries, respectively. The area of countries was retrieved from the infoplease.com website [15].

### Studied variables

Studied variables included the percentage of estimated helmet-wearing rate, the effectiveness of helmet law enforcement, the effectiveness of speed law enforcement, estimated road traffic deaths rate per 100,000 population, percentage of motorized 2–3 wheelers death, country population, gross national income (GNI) per capita in US dollars, number of registered vehicles, and percentage of motorized 2–3 wheelers in each country.

The percentage of motorized 2–3 wheelers death included all riders (drivers or passengers). The percentage of estimated helmet-wearing rate in our study included all riders. However, if data was not available on all riders, we used instead the reported percentage of estimated helmet-wearing rate of drivers. Information on the overall effectiveness levels of both helmet law enforcement and speed limit enforcement were scored on a scale of 0 to 10, where 0 is “not effective” and 10 is “highly effective” based on professional consensus in each country.

### Data entry

Data collected during all studied years were coded and entered into the MS Excel program in two formats: vertical data format (the same variables in all studied years were entered into a single column in order of years with an added year variable), and horizontal data format (each variable in each studied year entered into a separate column). Data were rechecked for accuracy and consistency and exported into SPSS for analysis.

### Calculations

The population density was calculated by dividing the total population by country area (number of people/mile square). The number of motorized 2–3 wheelers was calculated by multiplying the percentage of motorized 2–3 wheelers by the total number of registered vehicles. Motorized 2–3 wheelers death rate was calculated by multiplying the percentage of motorized 2–3 wheelers death by the estimated traffic road death rates per 100,000 population. Vehicle per person ratio was calculated by dividing the total number of registered vehicles by the total population. Motorized 2–3 wheelers per person ratio was calculated by dividing the total number of motorized 2–3 wheelers by total population.

### Statistical analysis

Mortality rates were highly skewed to the right. Accordingly, median (interquartile range, IQR) were used in reporting the data. We used a mixed linear model (MLM) to assess the factors affecting motorized 2–3 wheelers death rates over time. Death rates were transformed to a normal distribution to fulfill the requirements of the MLM. Log transformation had the best normal distribution over time and within each year and was used as the outcome variable.

The MLM analyses data of repeated measures (years) of each country (subject) separately, taking into account both the slope and intercept of each linear line of a country (within-subjects correlation). Studying the slope has an important advantage of addressing missing data and the nonlinear relationship between different factors. MLM assumes a normal distribution of the outcome (dependent) variable. The logarithmic transformation of death rate was the dependent variable of the MLM model, while independent covariates do not need to have a normal distribution which can be ordinal, continuous, or categorical data.

The used MLM model was a strict unstructured, main-effects model with repeated measures. It included a fixed effect, type III sum of squares error (due to the unbalanced data), and random effects for the independent variables (factors and covariates). These strict requirements assume that the variance of each studied year and the covariance (correlation) between the studied independent factors are different. The change of the outcome-dependent variable (death rate) was studied over time by entering the studied years as a categorical factor (factor = year) while independent variables as covariates. These included continuous variables (population density, GNI per capita, vehicle per person ratio, and motorized 2–3 wheelers per person ratio) and ordinal variables (speed law enforcement (0–10) and helmet law enforcement (0–10)). We tested different interactions in the model, excluded non-significant and included significant interactions in the final MLM model. Accordingly, the interaction between vehicle per person ratio and motorized 2–3 wheelers per person ratio was added to the final main effects model.

After achieving the results of the final MLM model, several univariate post hoc analyses were performed to explain our findings. Spearman rank correlation test was used to study the correlation between different continuous or ordinal variables. Wilcoxon signed-rank test was used to compare the continuous or ordinal data of two dependent groups. Friedman test was used to compare the continuous or ordinal data of more than two dependent groups. Mann–Whitney U test was used to compare the continuous or ordinal data of two independent groups, while Kruskal–Wallis test was used to compare the continuous or ordinal data of more than two independent groups. Data were analyzed with the IBM SPSS Statistics version 26 (SPSS Inc, Chicago, IL, USA). A *p*-value of less than 0.05 was accepted as statistically significant.

## Results

**Fig. 1:**
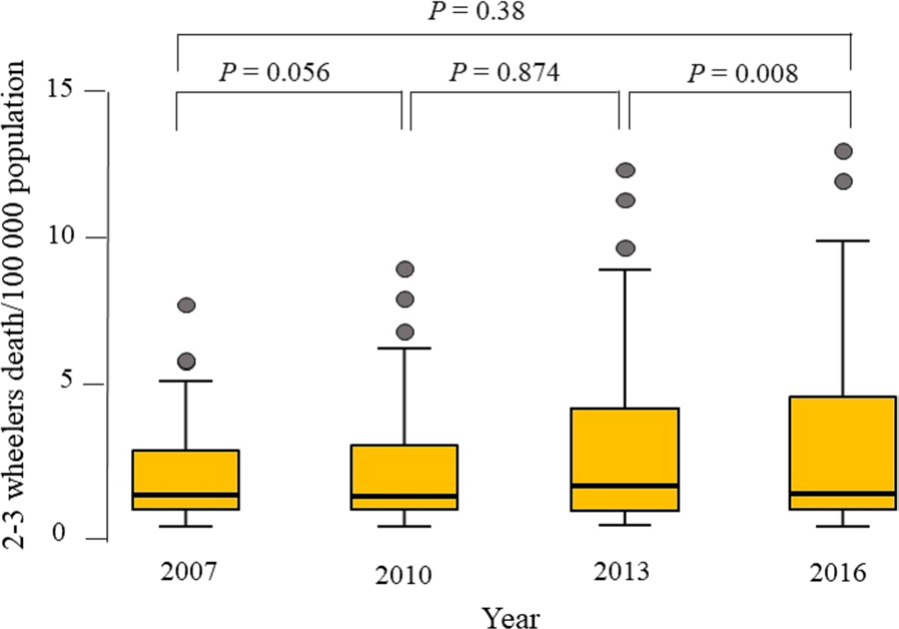
Box-and-whiskers plot of motorized 2–3 wheelers death rate per 100,000 population of years 2007–2016. The box resembles the 25th percentile and the 75th percentile Interquartile Range (IQR), while the line within the box resembles the median. Black circles represent the outliers. *p*-value = Friedman test for comparison of more than two dependent groups and Wilcoxon signed-rank test for comparison of two dependent groups

Table 1 shows the results of the mixed linear model. The model showed factors that affected the log transformation of motorized 2–3 wheelers death rates. The significant factors were GNI (*p* = 0.025), motorized 2–3 wheelers per person ratio (*p* < 0.0001), percentage of helmet wearing rate (*p* = 0.046), and the interaction between vehicle per person ratio and motorized 2–3 wheelers per person ratio (*p* = 0.016). No significant differences in mortality were seen over time. This was confirmed using post hoc analysis (*p* = 0.38, Friedman test) (Fig. 1). Although non-significant, the global mean motorized 2–3 wheelers death rates increased from 2.37/100,000 population to 3.23/100,000 population over the studied decade (a relative ratio of 1.36).

**Table 1:** Linear mixed effect model of factors affecting log transformation of motorized 2–3 wheelers death rate globally over 2007–2016

Variable	Estimate	SE	*t*-value	*p*-value	LL 95% CI	UL 95% CI
Year 2007	0.017	0.130	0.133	0.896	− 0.266	0.301
Year 2010	0.059	0.100	0.591	0.558	− 0.144	0.262
Year 2013	− 0.003	0.083	− 0.041	0.968	− 0.170	0.163
Density of population	5.779^−5^	5.536^−5^	1.044	0.302	− 5.370^−5^	0.169^−3^
GNI	− 1.220^−5^	5.239^−6^	− 2.329	0.025	− 2.280^−5^	− 1.602^−6^
Vehicle per person ratio	0.545	0.489	1.114	0.268	− 0.427	1.517
Motorized 2–3 wheelers/person ratio	7.851	1.725	4.552	0.000	4.433	11.270
Enforcement of speed	− 0.039	0.023	− 1.666	0.103	− 0.087	0.008
Enforcement of helmet	0.011	0.021	0.531	0.599	− 0.031	0.053
Percentage of helmet wearing rate	− 0.004	0.002	− 2.047	0.046	− 0.008	− 7.898^−5^
Vehicle/person ratio * motorized 2–3 wheelers/person ratio	− 4.792	1.953	− 2.454	0.016	− 8.667	− 0.916
Intercept	0.637	0.249	2.558	0.012	0.143	1.131

SE, standard error; LL, lower limit; CI, confidence interval; UL, upper limit

**Fig. 2:**
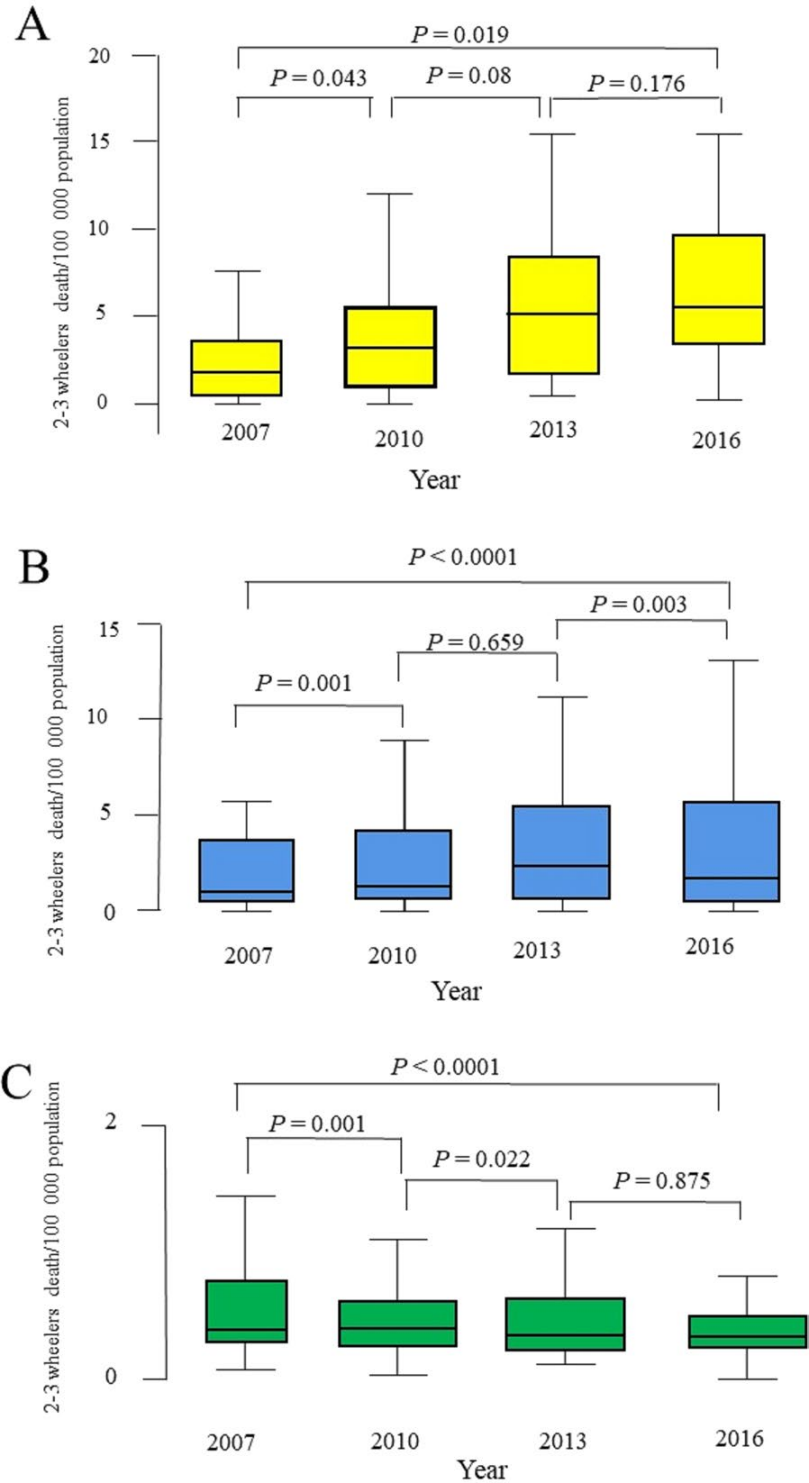
**A**–**C**: Box-and-whiskers plot of motorized 2–3 wheelers death rate per 100,000 population of years 2007–2016 by the level of income of countries (**A** = low-income countries, **B** = middle-income countries, and **C** = high-income countries). The box resembles the 25th percentile and the 75th percentile Interquartile Range (IQR), while the line within the box resembles the median. *p*-value = Friedman test for comparison of more than two dependent groups and Wilcoxon signed-rank test for comparison of two dependent groups

Nevertheless, there was a significant difference in the death rates depending on the income level. There was a statistically significant increase in mortality rate in low-income countries over time (*p* = 019, Friedman test, Fig. 2A). The mean death rate in the low-income countries increased from 3.01/100,000 population to 7.60/100,000 population over the studied decade (a relative ratio of 2.52). Furthermore, there was a statistically significant increase in the mortality rate in the middle-income countries over time (*p* < 0.0001, Friedman test, Fig. 2B). The mean death rate in the middle-income countries increased from 2.65/100,000 population to 3.86/100,000 population over the studied decade (a relative ratio of 1.46). The increase was significant between years 2007 and 2010 (*p* = 0.001, Wilcoxon signed-rank test). However, there was a significant drop between 2013 and 2016 (*p* = 0.003, Wilcoxon signed-rank test). There was a significant decrease in death rate in the high-income countries over time (*p* < 0.0001, Friedman est, Fig. 2C). The mean death rate in the high-income countries decreased from 1.92/100,000 population to 1.38/100,000 population over the studied decade (a relative ratio of 0.72).

**Fig. 3:**
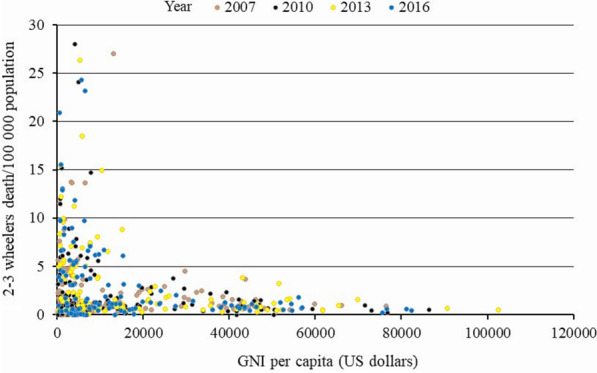
Correlation (scatter plot) between motorized 2–3 wheelers death rate per 100,000 population and GNI per capita in US dollars within each years of 2007, 2010, 2013, and 2016

**Fig. 4:**
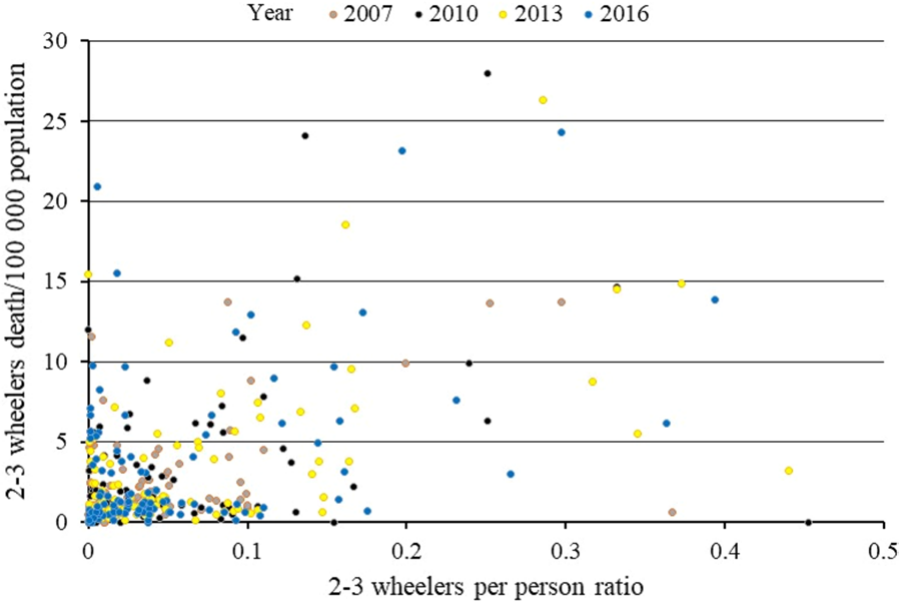
Correlation (scatter plot) between motorized 2–3 wheelers death rate per 100,000 population and motorized 2–3 wheelers/person ratio within each years of 2007, 2010, 2013, and 2016

**Fig. 5:**
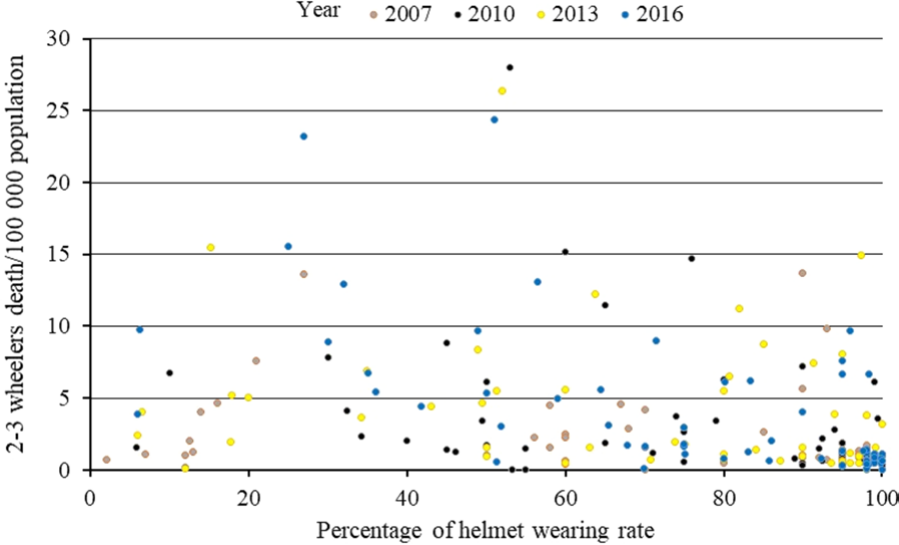
Correlation (scatter plot) between motorized 2–3 wheelers death rate per 100,000 population and percentage of helmet wearing rate within each years of 2007, 2010, 2013, and 2016

**Fig. 6:**
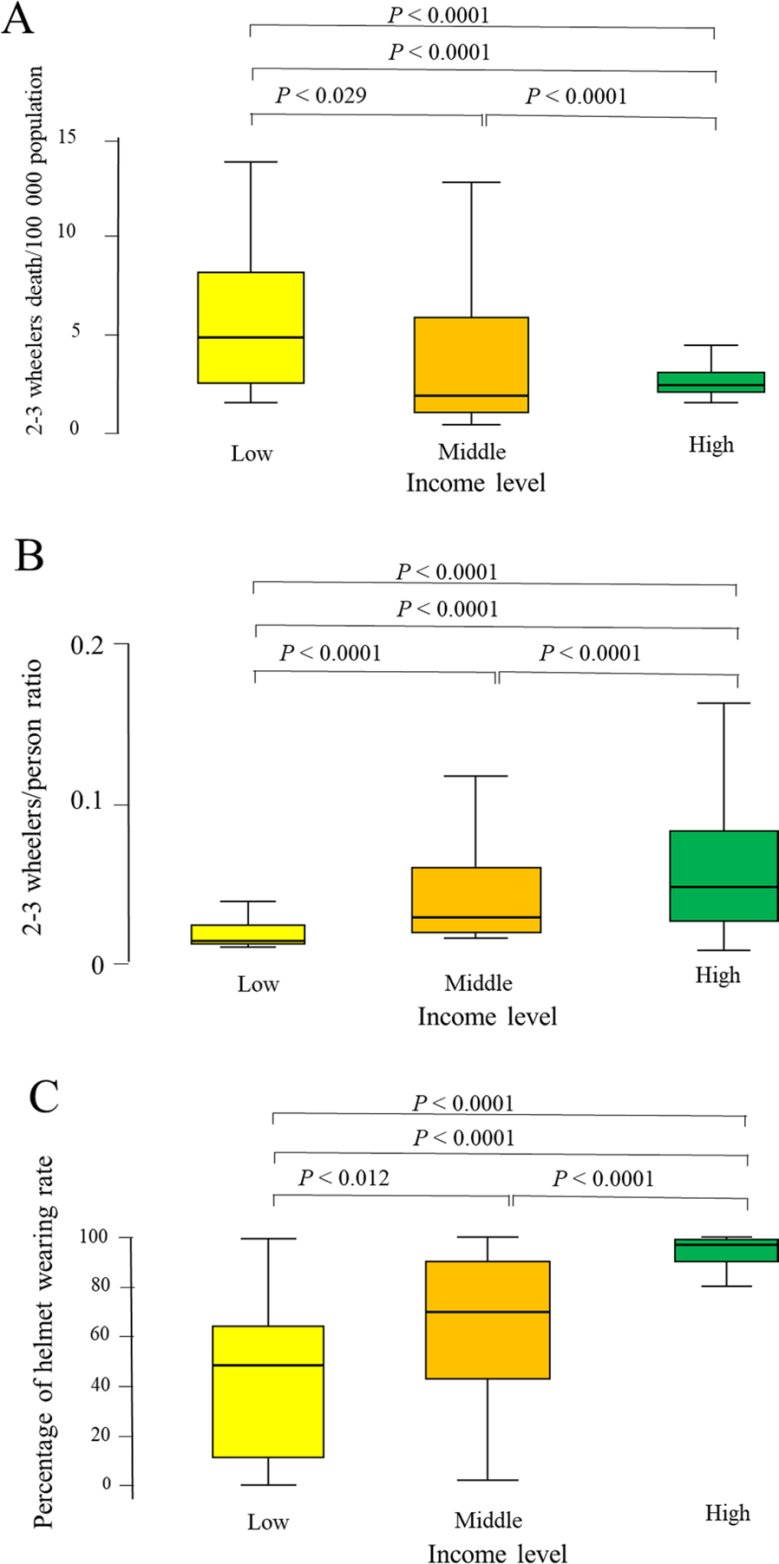
Box-and-whiskers plot of motorized 2–3 wheelers death rate per 100,000 population (**A**), motorized 2–3 wheelers/person ratio (**B**), and percentage of helmet wearing rate (**C**) of years 2007–2016 by the level of income of countries. The box resembles the 25th percentile and the 75th percentile Interquartile Range (IQR), while the line within the box resembles the median. *p*-value = Mann–Whitney *U* test for comparison of two independent groups and Kruskal–Wallis for comparison of more than two independent groups

Table 2 shows the correlations between 2–3 wheelers per person ratio, helmet usage, and GNI, with death rate (Table 2). All these correlations were highly significant within 2007, 2010, 2013, and 2016 (Figs. 3, 4, 5). The post hoc comparisons between low-income, middle-income, and high-income countries confirmed these correlations. There was a highly significant difference in the death rate, 2–3 wheelers per person ratio, and percentage of helmet-wearing rate between these countries. Death rate decreased, 2–3 wheelers per person ratio increased, while helmet usage increased with increased income (Fig. 6).

**Table 2:** Spearman rank correlations between the significant factors that affected motorized 2–3 wheelers death rate globally over 2007–2016

Variable	2–3 Wheelers per person ratio		Percentage of helmet wearing rate		GNI per capita	
	rho	*p*-value	rho	*p*-value	rho	*p*-value
*Year 2007*						
Motorcycle 2-to-3 wheelers death rate	0.408	*p* < 0.0001	− 0.342	*p* = 0.019	− 0.048	*p* = 0.610
GNI	0.494	*p* < 0.0001	0.390	*p* = 0.002	–	
*Year 2010*						
Motorcycle 2-to-3 wheelers death rate	0.35	*p* < 0.0001	− 0.389	*p* = 0.003	− 0.263	*p* = 0.004
GNI	0.443	*p* < 0.0001	0.538	*p* < 0.0001	–	
*Year 2013*						
Motorcycle 2-to-3 wheelers death rate	0.382	*p* < 0.0001	− 0.422	*p* = 0.001	− 0.366	*p* < 0.0001
GNI	0.420	*p* < 0.0001	0.669	*p* < 0.0001	–	
*Year 2016*						
Motorcycle 2-to-3 wheelers death rate	0.324	*p* = 0.001	− 0.661	*p* < 0.0001	− 0.312	*p* < 0.0001
GNI	0.294	*p* < 0.0001	0.732	*p* < 0.0001	–	

GNI, Gross National Income/capita (US dollars)

## Discussion

Our study has shown that there was no significant reduction in the global motorized 2–3 wheelers death rates over the studied period which has even increased by a relative ratio of 1.36. The UN target was not met. The death rate increased because of the increase in the motorized 2–3 wheelers and was reduced by helmet compliance and wealth. Nevertheless, there was a significant difference in the death rates between the three levels of income of countries with a relative ratio increase of 2.52 in the low-income countries and 1.46 in the middle-income countries, while it decreased by relative ratio of 0.72 in the high-income countries. These differences in the direction of change between the three levels explain why there was no overall global significant difference in the death rates when combined together. Other studies showed that the UN target was not met [16–18] and even suggested that mortality from motorized 2–3 wheelers would even increase globally by 11% over the coming 10 years [19].

The increased death rates were related to the increased motorized 2–3 wheelers in our study which was supported by others [14, 20, 21]. The use of motorized 2–3 wheelers increase the chances of getting a job in poor countries. Understandably, their increased number and travel time on unsafe roads would increase the RTCs [4, 22–24]. This would be even worse if safety rules were not followed like using helmets. Helmet compliance significantly reduced the death rate of the 2–3 wheeler drivers and riders in our study. Helmets reduce the impact of head injuries which have high mortality in the 2–3-wheeler-related injuries [8, 14]. Low GCS, indicating severe head injury, is one of the most important factors predicting mortality in RTCs [25–27].

The increased GNI reduced the overall mortality overtime in our study. Overall mortality depends on multiple factors that are correlated to each other. Finding a significance single univariate correlation with mortality is not enough to indicate that it is a predictor of mortality because it can be a confounder of another factor. Although the GNI significantly increased the number of 2–3 wheelers, it was highly correlated with an increase in helmet use in the population within each studied year. It is also possible that motorcycle drivers in high-income countries respect the speed limits and follow traffic regulations more strictly which reduces collision and death rates. Nevertheless, the mixed linear model is a strong model which depends on the slope of change within each country, can compensate for missing data, and will consider all these interactions to properly define predictors of mortality. Several previous studies showed that the increase in motorized 2–3 wheeler per person ratio is associated with initial rise of GNI. This ratio declines later on when the GNI increases more. Cars will be preferred at this stage because they are safer and more comfortable [6, 21, 28–30]. The increased GNI is also related to the effective implementation of road safety regulations (including helmet law), and improvement in medical care [28, 31].

## Limitation of the study

Our study has certain limitations. *First*, our analysis depended on the WHO reports. The availability and accuracy of this data can be affected by the political agenda and the health informatics infrastructure. The World Bank highlighted a profound difference between government-reported road deaths and WHO-estimated road deaths, with under-reporting of 84% in the low-income countries, 51% in middle-income countries, and 11% in high-income countries, [5]. We have used the estimated death rates of the WHO reports as it is more accurate. Nevertheless, this carries the risk of theoretical assumptions and modelling. *Second,* some important factors related to road deaths are not included in the analysis like the driver behavior, age, gender, drug/alcohol use, educational level, riding experience, and using visibility aids [8, 32]. Our study is based on a country level and not on individual levels. Factors that apply to a particular person are difficult to quantify on a country level. *Third*, publishing WHO reports takes up to 3 years. The report for the recent 3 years has not yet been published and is not included in our current analysis. This is most properly related to the COVID-19 pandemic where more WHO resources were used to address the pandemic challenges. We are awaiting this global report as it may highlight the impact of COVID-19 pandemic on the RTC safety [26, 33]. *Forth*, the GNI is a single collective factor with varying effects at different stages of the economic development. Although GNI in general was associated with the reduced motorized 2–3 wheelers deaths in our study, the rise of GNI at early stages would increase motorized 2–3 wheeler per person ratio while the law enforcement of safety regulations usually comes after the initial increase of mortality. *Finally,* data on other safety devices that can reduce motorcycle-related deaths like the Anti-Lock Brake Systems which may enable the driver to stop within a short distance [34, 35] were missing. We were limited by the available data in the WHO reports, and these data were not available.

## Conclusions

Global mortality of motorized 2–3 wheelers has increased by a relative ratio of 1.36 over a recent decade. The UN target of reducing death was not met. The increase was related to the increase in motorized 2–3 wheelers per person ratio and economic inequiety which has to be addressed globally. The economic global gap significantly impacts the mortality rates of motorized 2–3 wheelers.

## Data Availability

Original data are published by the WHO and available on the website references [1, 9, 10, 13].

## Abbreviations

CI: Confidence interval
GNI: Gross national income
LMICs: Low- and middle-income countries
LL: Lower limit
MIC: Middle-income countries
MLM: Mixed linear model
RTC: Road traffic collision
SE: Standard error
SPSS: Statistical package for the social sciences
UL: Upper limit
UN: United Nations
US: United States
USA: United States of America
WHO: World Health Organization
